# Candidemia in ICU Patients: What Are the Real Game-Changers for Survival?

**DOI:** 10.3390/jof11020152

**Published:** 2025-02-17

**Authors:** Bianca Leal de Almeida, Caroline Agnelli, Thaís Guimarães, Teresa Sukiennik, Paulo Roberto Passos Lima, Mauro José Costa Salles, Giovanni Luís Breda, Flavio Queiroz-Telles, Ana Verena Almeida Mendes, Luís Fernando Aranha Camargo, Hugo Manuel Paz Morales, Viviane Maria de Carvalho Hessel Dias, Afonso Rafael da Silva Junior, João Nóbrega de Almeida Junior, Camila de Melo Picone, Evangelina da Motta Pacheco Alves de Araújo, Edson Abdala, Flávia Rossi, Arnaldo Lopes Colombo, Marcello Mihailenko Chaves Magri

**Affiliations:** 1 Instituto do Câncer do Estado de São Paulo, School of Medicine, Hospital Infection Control and Infectious Diseases Service, University of São Paulo, São Paulo 01246-000, Brazil; bianca.almeida@hc.fm.usp.br (B.L.d.A.); edson.abdala@hc.fm.usp.br (E.A.); 2 Division of Infectious Diseases, Department of Medicine, Escola Paulista de Medicina, Universidade Federal de São Paulo, São Paulo 04024-002, Brazil; agnellicarol@gmail.com (C.A.); prpassoslima@hotmail.com (P.R.P.L.); sallesinfecto@gmail.com (M.J.C.S.); luis.camargo@einstein.br (L.F.A.C.); jnaj99@gmail.com (J.N.d.A.J.); arnaldolcolombo@gmail.com (A.L.C.); 3 Hospital das Clínicas da Faculdade de Medicina USP (FMUSP), São Paulo 05403-010, Brazil; thais.guimaraes@hc.fm.usp.br (T.G.); camila.picone@hc.fm.usp.br (C.d.M.P.); 4 Hospital do Servidor Público Estadual de São Paulo, São Paulo 04039-000, Brazil; 5 Santa Casa de Misericórdia de Porto Alegre, Porto Alegre 90050-170, Brazil; teresa@santacasa.org.br; 6 Santa Casa de Misericórdia de São Paulo, São Paulo 01221-010, Brazil; 7 Departamento de Saúde Coletiva, Universidade Federal do Paraná, Curitiba 81531-990, Brazil; giovanni.breda@hc.ufpr.br (G.L.B.);; 8 Hospital Nossa Senhora das Graças, Curitiba 80810-040, Brazil; carvalhohdias@gmail.com; 9 Hospital São Rafael, Salvador 41253-190, Brazil; ana.verena@rededor.com.br; 10 Escola Bahiana de Medicina e Saúde Pública (EBMSP), Salvador 40290-000, Brazil; 11 Instituto D’OR de Pesquisa e Ensino-(IDOR), Salvador 41253-190, Brazil; 12 Hospital Israelita Albert Einstein, São Paulo 05652-900, Brazil; 13 Hospital Erasto Gaertner, Curitiba 81520-060, Brazil; moraleshmp@gmail.com; 14 Laboratório de Microbiologia da Divisão de Laboratório Central, Pathology Department, Hospital das Clínicas da Faculdade de Medicina USP (FMUSP), São Paulo 05403-010, Brazil; afonso.rafael@hc.fm.usp.br (A.R.d.S.J.); evangelina.araujo@hc.fm.usp.br (E.d.M.P.A.d.A.); f.rossi@hc.fm.usp.br (F.R.); 15 Department of Infectious and Parasitic Diseases, School of Medicine, University of São Paulo, São Paulo 05508-220, Brazil

**Keywords:** candidemia, catheter removal, cancer, intensive care unit, echinocandins

## Abstract

Candidemia infection remains a critical challenge in intensive care units (ICUs), with high morbidity and mortality rates despite advances in therapeutic practices. This multicenter prospective surveillance study assessed the epidemiology, clinical management, and mortality predictors of candidemia in critically ill patients across two periods (2010–2012 and 2017–2018) in 11 tertiary hospitals in Brazil. Among 314 ICU patients with candidemia, the overall mortality rate was 60.2%, with no significant reduction over time (58.8% vs. 62.6%, *p* = 0.721). *Candida albicans* was the predominant pathogen (43.6%), followed by *C. tropicalis* (20%) and *C. glabrata* (13.7%). The use of echinocandins increased significantly in the second period (21.1% to 41.7%, *p* < 0.001); however, 70% of patients still did not receive these agents as first-line therapy. Catheter removal due to candidemia was performed in only 52.1% of cases but was associated with improved 30-day survival (*p* < 0.001). Multivariate analysis identified cancer, inadequate treatment, and vasoactive drug use as independent predictors of mortality. Our findings underscore persistent gaps in adherence to guidelines, particularly regarding timely echinocandin initiation and catheter removal. Strengthening therapeutic strategies focused on these key interventions is essential to improving outcomes for ICU patients with candidemia.

## 1. Introduction

Candidemia is the most significant invasive fungal disease in critically ill patients and ranks among the ten most frequent healthcare-associated bloodstream infections in intensive care units (ICUs) [1,2,3]. The incidence of candidemia among critically ill patients ranges from 5.5 to 7 episodes per 1000 ICU admissions in recent reports [4,5].

The epidemiology of candidemia and its management strategies in ICUs have undergone notable changes in recent years, as documented in contemporary studies and aligned with evidence-based recommendations from relevant guidelines [6,7,8]. However, despite advances in therapeutic approaches, candidemia patients continue to experience high morbidity and mortality rates, leading to poor outcomes in the ICU [9,10,11,12].

Understanding the epidemiology [13] and antifungal susceptibility of *Candida* spp. is essential for effective clinical management and the development of targeted treatment protocols. In Brazil, antifungal resistance patterns remain underexplored. In a systematic review, Hamburger et al. (2024) analyzed publications from 2017 to 2023, identifying 16 studies that met the inclusion criteria and which comprised 2305 episodes of candidemia. The predominant species were *C. albicans*, *C. parapsilosis*, and *C. tropicalis*, with notable proportions of *C. glabrata*. Resistance to echinocandins was rare (1.5%, 6/396 isolates), whereas fluconazole resistance showed significant variability, ranging from 0% to 43% [14].

We conducted a multicenter prospective surveillance study to evaluate trends in epidemiology and clinical practices related to the management of critically ill patients with candidemia admitted to ICUs in tertiary hospitals in Brazil. The primary objective was to investigate mortality rates and identify predictors of 30-day mortality. Secondary objectives included comparing the epidemiology, therapeutic management, and predictors of mortality across two distinct periods.

## 2. Materials and Methods

### 2.1. Surveillance

This study merged two databases collected during epidemiological studies conducted by our group [11,12,15,16,17]. The databases included clinical and laboratory information regarding all episodes of candidemia sequentially documented in patients admitted to the ICUs of eleven tertiary hospitals in Brazil across two periods: 2010–2012 (period 1) and 2017–2018 (period 2). The participating centers were located across various Brazilian states and included the following: Hospital das Clínicas da Faculdade de Medicina da Universidade de São Paulo (FMUSP), São Paulo; Hospital São Paulo, Universidade Federal de São Paulo, São Paulo; Hospital do Servidor Público Estadual de São Paulo, São Paulo; Santa Casa de Misericórdia de Porto Alegre, Rio Grande do Sul; Santa Casa de Misericórdia de São Paulo, São Paulo; Universidade Federal do Paraná, Curitiba, Paraná; Hospital São Rafael, Salvador, Bahia; Hospital Israelita Albert Einstein, São Paulo; Hospital Erasto Gaertner, Curitiba, Paraná; and Hospital Nossa Senhora das Graças, Curitiba, Paraná. Each medical center had a local investigator trained to select cases and collect data on all adult patients who were sequentially admitted with candidemia using a standard clinical form and dictionary of terms. Our aim was to determine mortality rates among patients with candidemia in critical condition, as well as to assess prognostic factors associated with 30-day mortality. We compared epidemiology, therapeutic management, and mortality predictors between periods 1 and 2 to investigate the historical trends of candidemia in the ICU.

Local investigators were asked to check reports of candidemia provided by the microbiology laboratory of their medical centers daily. All cases of candidemia had their clinical chart reviewed with the aid of a standard clinical form complemented by a dictionary of terms. Clinical variables included demographics, underlying conditions, clinical manifestations, time to initiation of antifungal therapy, antifungal therapy regimens, catheter management, date and reason for central venous catheter (CVC) removal, and 30-day crude mortality. To assess the severity of the cases, the APACHE score was used in the first period and the sequential evaluation of organ failure (SOFA) was used in the second period. Septic shock was defined as hypotension not responding to fluid therapy and requiring vasoactive agents. Thus, to standardize the data across the two periods, we analyzed the use of vasoactive drugs as a predictor of severity based on the literature [18].

### 2.2. Case Definitions

Candidemia was defined as the incident isolation of *Candida* spp. from an appropriately collected blood culture [19,20]. Candidemia occurring >30 days after the incident isolation was defined as a new case. Patients were considered adults if they were >18 years old. Fever was defined as body temperature >37.7 °C, and hypotension was defined as systolic blood pressure (BP) < 90 mmHg and mean arterial pressure < 70 mmHg or a reduction in BP of >40 mmHg from baseline. Renal injury was defined as a serum creatinine level exceeding 1.5 mg/dL at any point within the past three months. Dialysis was considered if the patient underwent the procedure within two weeks prior to the onset of candidemia. Neutropenia was defined as an absolute neutrophil count below 500/mm^3^. Inadequate treatment was defined as the failure to remove the CVC and the lack of use of an echinocandin [21,22].

### 2.3. Microbiology

*Candida* isolates were initially identified to the species level in the local laboratories and subsequently sent to the Special Mycology Laboratory (LEMI) at the Universidade Federal de São Paulo for confirmation. During the first study period, identification at the central laboratory was performed using microscopic evaluation on cornmeal Tween 80 agar combined with biochemical analysis via the ID32C system (BioMérieux SA, Marcy l’Étoile, France). In the second period, all *Candida* isolates were identified using Matrix-Assisted Laser Desorption/Ionization–Time-of-Flight Mass Spectrometry (MALDI-TOF MS) Biotyper System, Bruker (Billerica, MA, USA). Antifungal susceptibility tests were performed by broth microdilution assays using the Clinical & Laboratory Standards Institute (CLSI) methodology.

### 2.4. Data Analysis

Results for quantitative variables were described as median and interquartile ranges. Categorical variables were described using frequency and percentage. For the univariate and multivariate analysis of factors associated with death within 30 days, logistic regression models were adjusted considering death within 30 days. Variables that showed statistical significance (*p* < 0.05) in the univariate analysis were included in the multivariate model. To adjust this model, a stepwise backward approach was used (with a probability of 0.10 for removing variables). The significance of the variables was analyzed using the Wald test, and the estimated measure of association was the odds ratio with respective 95% confidence intervals.

To compare the different periods (2010–2012 vs. 2017–2018), Student’s *t*-test for independent samples or the non-parametric Mann–Whitney test were used to check continuous variables. Categorical variables were analyzed using Fisher’s exact test or the chi-square test, where appropriate. For a description of the survival time in the two periods, Kaplan–Meier curves were used. The comparison of curves was made using the Gehan–Breslow–Wilcoxon test. Values of *p* < 0.05 were considered statistically significant. Data were analyzed using the computer program Stata/SE v.14.1. Stata Corp LP, College Station, TX, USA.

Kaplan–Meier survival curves were constructed to assess the impact of catheter removal on patient outcomes. The analysis included three groups: catheter removal specifically due to candidemia, absence of catheter removal, and catheter removal for reasons unrelated to candidemia. The null hypothesis—that there is no difference in survival curves between the classifications of the variable (yes or no)—was tested against the alternative hypothesis of differing survival curves. Statistical significance was evaluated using the Wilcoxon test, with a threshold of *p* < 0.05.

## 3. Results

A total of 314 ICU patients with candidemia were evaluated over two periods: 199 patients in period 1 and 115 in period 2. There was a similar distribution between genders (161 female, 51.3%), with a median age of 61 years. The main comorbidities included kidney injury (134, 42.6%), cancer (96, 30.6%), solid tumors (78, 24.8%), and diabetes mellitus (78, 24.8%). Major risk factors for candidemia were mechanical ventilation (222, 70.7%), hypotension (173, 55%), the use of vasoactive drugs (171, 54.4%), surgery (166, 52.8%), presence of a CVC (148, 52.1%), and kidney failure (102, 32.4%).

Regarding therapeutic management, fluconazole was the most frequently used antifungal agent (127, 40.4%), while echinocandins were administered to only 90 patients (28.7%). Echinocandin use was associated with lower mortality in the univariate analysis. The overall mortality rate among critically ill ICU patients with candidemia was 60.2%. Mortality rates by species were as follows: *C. albicans* (62.7%), *C. tropicalis* (57.1%), *C. glabrata* (55.8%), *C. parapsilosis* (54.7%), and *C. krusei* (61.5%). No statistically significant differences were observed among these species-specific mortality rates.

The baseline characteristics, prognostic factors, and univariate analysis are detailed in Table 1.

The most frequently isolated Candida species was *C. albicans* (137, 43.6%), followed by *C. tropicalis* (20%) and *C. glabrata* (13.7%). *C. parapsilosis* and *C. krusei* accounted for 13.4% and 4.1% of cases, respectively. Susceptibility testing was performed on 118 isolates, including *C. albicans* (56 isolates), *C. tropicalis* (27), *C. glabrata* (16), *C. parapsilosis* (14), and *C. krusei* (5). The MICs for *C. glabrata* ranged from 0.5 to 8.0 µg/mL. No resistance to azoles was observed in *C. albicans*, *C. tropicalis*, or *C. parapsilosis*, and no resistance to echinocandins was detected in any of the tested species. Deep-seated infections were diagnosed in 23 patients (7.3%) with candidemia.

For multivariate analysis, the following variables were included: age, cancer, inadequate treatment, surgery, use of vasoactive drugs, treatment with echinocandins, and non-treatment with fluconazole. Multivariate analysis identified cancer (OR = 2.06; 95% CI, 1.13–3.73; *p* = 0.018), inadequate treatment (OR = 2.27; 95% CI, 1.56–4.90; *p* = 0.001), and the use of vasoactive drugs (OR = 3.75; 95% CI, 2.21–6.38; *p* < 0.001) as significant mortality risk factors (Table 2).

The comparison between the first and second periods showed mortality rates of 58.8% vs. 62.6%, respectively, with *p* = 0.721. For clinical management, we observed greater use of echinocandins in period 2: 21.1% in period 1 vs. 41.7% in period 2, with *p* < 0.001. However, the adherence to catheter removal due to candidemia was 50.6% in the first period vs. 54.5% in the second period, with *p* = 0.544. It was also observed that patients in period 2 were more likely to present hypotension (96 (48.5%) vs. 77 (67%); *p* = 0.002), thus requiring vasoactive drugs more often (96 (49.7%) vs. 75 (65.2%); *p* = 0.009); patients in period 2 were also more frequently submitted to dialysis (56 (28.4%) vs. 46 (40%); *p* = 0.045) and had more prior hospitalizations (40 (20.9%) vs. 46 (40%); *p* < 0.001). Other differences in clinical presentation, epidemiological factors, and therapeutic management characteristics between the first (1) and second (2) periods are shown in Table 3.

**Figure 1:**
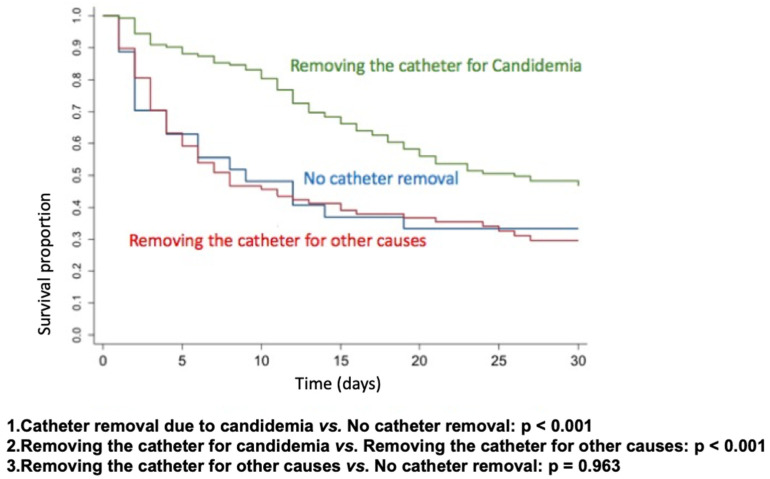
Kaplan–Meier survival curves based on CVC removal.

To better analyze the impact of catheter removal, we performed Kaplan–Meier survival curves for catheter removal due to candidemia vs. no catheter removal (*p* < 0.001), catheter removal for candidemia vs. catheter removal for other causes (*p* < 0.001), and catheter removal for other causes vs. no catheter removal (*p* = 0.963). Only catheter removal due to candidemia had a positive impact on 30-day survival (Figure 1).

## 4. Discussion

We conducted a prospective multicenter study to compare trends in the epidemiology, therapeutic practices in real-world settings, and mortality in a large cohort of adults with candidemia from the ICUs of public and private tertiary hospitals in Brazil, diagnosed over two distinct periods in the past decade. Despite the increased use of echinocandins, mortality rates remained high and showed no significant improvement over time, likely due to notable changes in the at-risk population, the severity of illness, delayed diagnosis, and therapeutic approaches. We also observed, through Kaplan–Meier survival curves, that catheter removal due to candidemia had a positive impact on 30-day survival.

In the past decade, guidelines for managing non-neutropenic patients with candidemia have emphasized several recommendations for best clinical practices, including the use of echinocandins as first-line therapy and the early removal of the CVC [6,7,8,23,24,25,26,27,28,29]. Despite these strong recommendations, our study found that approximately 50% of patients with candidemia did not have their CVC removed, around 70% did not receive echinocandins, and nearly 20% did not receive any antifungal therapy. These data indicate that many patients did not receive adequate antifungal treatment, either due to improper drug selection or delays in receiving the results of blood cultures. These findings contrast with those reported by Araújo et al. (2020) in a single-center study conducted in São Paulo, Brazil, which evaluated adherence to Brazilian guidelines for candidemia management. In that study, 69.5% of the 115 patients received antifungal treatment, a proportion similar to our findings. However, CVC removal was performed in 82.5% of cases and 60% of patients were started on echinocandins [24].

Robust data in the literature support the use of echinocandins as the drug of choice for the treatment of candidemia owing to reduced lethality in these patients [6,7,8,23,26,27,28,29,30]. In our series, only 28% of the patients in the intensive care unit had initial therapy performed with echinocandins. Despite strong recommendations for the use of echinocandins, their utilization remains limited. Even when examining the two periods separately, in the second period, around 60% of patients did not receive echinocandins as the first-line treatment.

Regarding etiology, we observed that *C. albicans* remains the primary causative agent, though there has been a shift in the proportion of candidemia cases caused by other *Candida* spp., such as *C. tropicalis* and *C. glabrata*, reinforcing the recommendation for the use of echinocandins as first-line therapy. These findings align with the results generated in other Brazilian studies [11,12,22,31]. Hamburger et al. (2024), in a systematic review, analyzed the distribution of *Candida* species in Brazilian patients with candidemia from 2017 to 2023. Among 7075 records screened, 16 studies met the inclusion criteria, encompassing 2305 episodes of candidemia. The predominant species were *C. albicans*, *C. parapsilosis*, and *C. tropicalis*, with a notable proportion of *C. glabrata* [14]. In the second period of our study, we observed a significant decrease in *C. parapsilosis* prevalence, from 16.6% in period 1 to 7.8% in period 2. This trend may be attributed to several factors: rigorous implementation of hand hygiene protocols; improved catheter management practices; selective pressure from antifungal use; a potential coincidence of the reduction in *C. parapsilosis* with an increase in other *Candida* spp., such as *C. albicans* or *C. glabrata*, driven by antifungal selective pressure; active surveillance and early identification; infection prevention training; and enhanced environmental and equipment disinfection.

Another crucial measure in managing patients with invasive candidiasis is the screening of deep-seated infection and focus control [6,7,8,18,23,27,32,33]. Bassetti et al. (2019) argue that source control is the most critical predictor of lethality in this population. In our study, only 7.3% of patients had *Candida* deep-seated infections at diagnosis. In most cases, catheter removal represents effective source control, either directly or indirectly, because even when the catheter is not the primary source of infection, persistent fungemia increases the likelihood that invasive devices become colonized by *Candida* spp., thereby perpetuating the infection [18,32]. Although several studies over the past decade have strongly recommended early CVC removal for all patients with candidemia, our findings indicate that the practice of catheter removal in candidemia management is still suboptimal. Nonetheless, we observed a positive impact on 30-day survival when comparing CVC removal due to candidemia with removal for other reasons at any time.

Agnelli et al. (2021) demonstrated in their multicenter study [22] that inadequate therapeutic management—defined as the absence of effective antifungal therapy and lack of CVC removal within 48 h—is an independent factor associated with 30-day mortality, consistent with our findings. These results highlight the importance of implementing a comprehensive set of measures to ensure optimal treatment for patients with candidemia. The same study reported that, in Brazil, the mortality rate of ICU patients with candidemia was approximately 20% higher than in Spain (65.1% vs. 39.7%). This disparity was directly linked to non-adherence to the recommended management practices, specifically the use of echinocandins and CVC removal within 48 h. Thus, suboptimal therapeutic management and partial adherence to standard recommendations for candidemia treatment are critical factors in explaining the higher mortality rates observed in Brazil compared to countries with stricter adherence to these guidelines [12,22].

This study has limitations. Although data were prospectively collected through routine laboratory surveillance, retrospective analyses were based on variables available across the eleven centers in both periods. Consequently, some critical aspects that could further elucidate the persistent high mortality and epidemiological trends, such as candidemia incidence, bacterial coinfection data, antifungal susceptibility patterns, catheter management details, time to blood culture positivity, notification time, availability of infectious disease specialists, and structural data, were not assessed. Nevertheless, this large multicenter series from public and private tertiary hospitals provides valuable insights into candidemia management in a developing country, highlighting that continuous trend monitoring is essential to guide individualized and adaptive management strategies.

## 5. Conclusions

In conclusion, our data indicate that the high mortality of critically ill ICU patients with candidemia is primarily attributable to inadequate therapeutic management, including a high proportion of untreated cases, low adherence to echinocandin use, and delayed CVC removal. Therefore, we advocate for reinforcing therapeutic management strategies in the ICU, prioritizing timely CVC removal and the early initiation of echinocandins as the preferred treatment to improve outcomes in this vulnerable population.

## Figures and Tables

**Table 1:** Clinical data and univariate analysis of 30-day mortality in 314 ICU patients with candidemia treated in 11 medical centers in Brazil across two time periods (2010–2012 and 2017–2018).

Variable	All Patients (314)	Alive (125)	Dead (189)	*p*	OR (CI 95%)
Age (mean, ST; years)	61.1 ± 18.0	58.1 ± 19.3	63.1 ± 16.8	0.016	1.02 (1.003–1.03)
Male	153 (48.7%)	55 (44%)	98 (51.9%)	0.174	1.37 (0.87–2.16)
Comorbidities					
Cancer	96 (30.6%)	29 (23.2%)	67 (35.4%)	0.022	1.82 (1.09–3.03)
Hematologic cancer	20 (6.3%)	9 (7.2%)	11 (5.8%)	0.625	0.80 (0.32–1.98)
Solid tumor	78 (24.8%)	21 (16.8%)	57 (30.1%)	0.008	2.14 (1.22–3.75)
Transplant	24 (7.6%)	10 (8%)	14 (7.4%)	0.847	0.92 (0.40–2.14)
Cardiac disease	86 (27.4%)	35 (28%)	51 (27%)	0.843	0.95 (0.57–1.58)
Lung disease	67 (21.3%)	20 (16%)	47 (24.8%)	0.060	1.75 (0.98–3.12)
*Diabetes mellitus*	78 (24.8%)	29 (23.2%)	49 (25.9%)	0.548	1.18 (0.69–1.99)
Kidney injury	134 (42.6%)	43 (34.4%)	91 (48.1%)	0.016	1.77 (1.11–2.82)
Chronic kidney disease	39 (12.4%)	11 (8.8%)	28 (14.8%)	0.117	1.80 (0.86–3.77)
Chronic kidney disease (Dialysis)	24 (7.6%)	8 (6.4%)	16 (8.4%)	0.501	1.35 (0.56–3.26)
Autoimmune disease	14 (4.4%)	8 (6.4%)	6 (3.1%)	0.190	0.49 (0.16–1.43)
Neurologic disease	87 (27.7%)	37 (29.6%)	50 (26.4%)	0.514	0.85 (0.51–1.40)
Surgery	166 (52.8%)	77 (61.6%)	89 (47%)	0.011	0.55 (0.35–0.87)
Abdominal surgery	83 (26.4%)	37 (29.6%)	46 (24.3%)	0.301	0.77 (0.46–1.27)
Burned	5 (1.59%)	4 (3.2%)	1 (0.5%)	0.104	0.16 (0.02–1.46)
Neutropenia	17 (5.4%)	5 (4%)	12 (6.3%)	0.366	1.64 (0.56–4.77)
Previous hospitalization	86 (27.3%)	33 (26.4%)	53 (28%)	0.705	1.10 (0.66–1.84)
Complications in ICU					
Parenteral nutrition	72 (22.9%)	30 (24%)	42 (22.2%)	0.752	0.92 (0.54–1.57)
Mechanical ventilation	222 (70.7%)	80 (64%)	142 (75.1%)	0.035	1.70 (1.04–2.78)
Kidney failure	102 (32.4%)	30 (24%)	72 (38%)	0.008	1.98 (1.20–3.29)
Hypotension	173 (55%)	45 (36%)	128 (67.7%)	<0.001	3.68 (2.29–5.93)
Vasoactive drug	171 (54.4%)	43 (34.4%)	128 (67.7%)	<0.001	4.18 (2.57–6.78)
Central venous catheter >48 h	298 (94.9%)	120 (96%)	178 (94.2%)	0.475	0.67 (0.23–1.99)
Central venous catheter removal because candidemia at any time (*n* = 284)	148 (52.1%)	70 (62.5%)	78 (45.3%)	0.005	0.50 (0.30–0.81)
Bacteremia	77 (24.5%)	33 (26.4%)	44 (23.2%)	0.466	0.82 (0.49–1.39)
Prior drug exposure					
Antibiotics **	300 (95.5%)	118 (94.4%)	182 (96.2%)	0.429	1.54 (0.53–4.51)
Prior antifungal ***	75 (23.8%)	31 (24.8%)	44 (23.2%)	0.698	0.90 (0.53–1.53)
Corticosteroids	130 (41.4%)	46 (36.8%)	84 (44.4%)	0.368	1.24 (0.78–1.99)
Chemotherapy *	22 (7%)	7 (5.6%)	15 (7.9%)	0.429	1.45 (0.58–3.67)
Etiology					
*C. albicans*	137 (43.6%)	51 (40.8%)	86 (45.5%)	0.411	1.21 (0.77–1.91)
*C. tropicalis*	63 (20%)	27 (21.6%)	36 (19%)	0.581	0.85 (0.49–1.49)
*C. parapsilosis*	42 (13.4%)	19 (15.2%)	23 (12.1%)	0.441	0.77 (0.40–1.49)
*C. glabrata*	43 (13.7%)	19 (15.2%)	24 (12.6%)	0.528	0.81 (0.42–1.55)
*C. krusei*	13 (4.1%)	5 (4%)	8 (4.2%)	0.919	1.06 (0.34–3.32)
Treatment ^†^					
No antifungal prescribed	64 (20.3%)	6 (4.8%)	58 (30.6%)	<0.001	8.78 (3.66–21.1)
Fluconazole	127 (40.4%)	50 (40%)	77 (40.7%)	0.153	0.71 (0.44–1.13)
Echinocandin	90 (28.7%)	47 (37.6%)	43 (22.8%)	0.004	0.46 (0.27–0.78)
Amphotericin	43 (13.6%)	15 (12%)	28 (14.8%)	0.980	0.991 (0.505–1.94)
Inadequate treatment	110 (35%)	28 (22.4%)	82 (43.3%)	0.001	2.73 (1.62–4.61)
Days between candidemia and treatment, *n* = 241—median (AIQ)	2 (3)	3 (4)	2(2)	0.006	0.87 (0.79–0.96)
Days of Treatment *n* = 248—median (AIQ)	10 (12)	14 (11)	6 (11)	<0.001	0.88 (0.85–0.92)
Deep-seated infections	23 (7.3%)	10 (8%)	13 (6.8%)	0.995	1.00 (0.42–2.37)
30-day crude mortality	189 (60.2%)	-	-	-	-

Logistic regression model and Wald test, *p* < 0.05; AIQ; interquartile range; ST: standard deviation; * in the last 14 days; ** in the last two days; *** in the last 30 days; ^†^ inadequate treatment was defined as the failure to remove the central venous catheter (CVC) and the lack of use of an echinocandin. One patient used voriconazole as treatment.

**Table 2:** Multivariate analysis of 30-day mortality in 314 ICU patients with candidemia treated in 11 medical centers in Brazil across two time periods (2010–2012 and 2017–2018).

Variable	*p* *	OR	CI 95%
Age	0.139	1.01	0.99–1.03
Cancer	0.018	2.06	1.13–3.73
Inadequate treatment	0.001	2.27	1.56–4.90
Surgery	0.086	0.62	0.36–1.07
Vasoactive drug	<0.001	3.75	2.21–6.38
Not treated with fluconazole but treated with echinocandin or amphotericin B	0.125	1.53	0.89–2.64

* Multivariate logistic regression model and Wald test, *p* < 0.05.

**Table 3:** Main clinical characteristics of 314 ICU patients with candidemia treated in 11 medical centers from Brazil across two time periods (2010–2012 versus 2017–2018).

Variable	Period		*p* *
	2010–2012 (*n* = 199)	2017–2018 (*n* = 115)	
Age (median; years)	61.5 ± 18.7	60.5 ± 16.8	0.623
Female	100 (50.3%)	61 (53%)	0.641
Comorbidities			
Cancer	59 (29.6%)	37 (32.2%)	0.703
Hematologic cancer	12 (6%)	8 (7%)	0.812
Solid tumor	47 (23.6%)	31 (27%)	0.588
Transplant	13 (6.5%)	11 (9.6%)	0.380
Cardiac disease	64 (32.2%)	22 (19.1%)	0.013
Lung disease	50 (25.5%)	17 (14.8%)	0.032
*Diabetes mellitus*	48 (24.4%)	30 (26.1%)	0.787
Kidney disease	78 (39.2%)	56 (48.7%)	0.124
Chronic kidney disease	18 (9%)	21 (18.3%)	0.021
Chronic dialysis	13 (6.5%)	11 (9.6%)	0.380
Autoimmune disease	8 (4.1%)	6 (5.2%)	0.778
Neurologic disease	61 (30.8%)	26 (22.6%)	0.150
Surgery	108 (54.8%)	58 (50.4%)	0.482
Abdominal surgery	51 (25.6%)	32 (27.8%)	0.692
Burned	3 (1.5%)	2 (1.7%)	1
Neutropenia	6 (3%)	11 (9.6%)	0.018
Complications in ICU			
Parenteral nutrition	43 (21.7%)	29 (25.4%)	0.487
days parenteral nutrition (median)	7.5 (1–113)	8 (1–150)	0.312
Mechanical ventilation	140 (70.4%)	82 (71.3%)	0.898
Days mechanical ventilation	9 (1–75)	8 (1–123)	0.695
Dialysis	56 (28.4%)	46 (40%)	0.045
Temperature	38.3 (36.6–40.4)	38 (35–39)	<0.001
Hypotension	96 (48.5%)	77 (67%)	0.002
Vasoactive drug	96 (49.7%)	75 (65.2%)	0.009
Central venous catheter	190 (95%)	108 (93.9%)	0.797
Bacteremia	51 (26.6%)	26 (23%)	0.585
Central venous catheter removal because candidemia at any time	88 (50.6%)	60 (54.5%)	0.544
Prior drug exposure			
Antibiotics	196 (98.5%)	104 (90.4%)	0.001
Prior antifungal	57 (28.9%)	18 (15.7%)	0.009
Corticosteroids	100 (53.2%)	30 (26.1%)	<0.001
Chemotherapy	13 (6.5%)	9 (7.8%)	0.654
Etiology			
*C. albicans*	83 (41.7%)	54 (46.9%)	0.409
*C. tropicalis*	40 (20.1%)	23 (20%)	0.999
*C. parapsilosis*	33 (16.6%)	9 (7.8%)	0.038
*C. glabrata*	26 (13%)	17 (14.8%)	0.734
*C. krusei*	9 (4.5%)	4 (3.4%)	0.789
Treatment			
Fluconazole	86 (43.2%)	41 (35.7%)	0.192
Echinocandin	42 (21.1%)	48 (41.7%)	<0.001
Amphotericin	35 (17.6%)	8 (7%)	0.010
Days between candidemia and start treatment	2 (0–68)	2 (−30 a 12)	0.361
Days of Treatment (median)	13 (0–66)	5.5 (0–29)	<0.001
Deep-site infection	14 (9.8%)	9 (7.8%)	0.664
30-day crude mortality	117 (58.8%)	72 (62.6%)	0.721
Previous hospitalization	40 (20.9%)	46 (40%)	<0.001

* Student’s *t*-test for independent samples or the non-parametric Mann–Whitney test were used for checking continuous variables. Categorical variables were analyzed using Fisher’s exact test or the chi-square test, when appropriate.

## Data Availability

The data presented in this study are available on request from the corresponding author due to privacy or ethical restrictions.

## References

[1] Timsit J.F., Ruppé E., Barbier F., Tabah A., Bassetti M. (2020). Bloodstream infections in critically ill patients: An expert statement. Intensive Care Med..

[2] World Health Organization (2022). WHO Fungal Priority Pathogens List to Guide Research, Development and Public Health Action. https://www.who.int/publications/i/item/9789240060241.

[3] Buetti N., Tabah A., Loiodice A., Ruckly S., Aslan A.T., Montrucchio G., Cortegiani A., Saltoglu N., Kayaaslan B., Aksoy F. (2022). Different epidemiology of bloodstream infections in COVID-19 compared to non-COVID-19 critically ill patients: A descriptive analysis of the Eurobact II study. Crit. Care..

[4] Lass-Flörl C., Kanj S.S., Govender N.P., Thompson GR 3rd Ostrosky-Zeichner L., Govrins M.A. (2024). Invasive candidiasis. Nat. Rev. Dis. Primers.

[5] Nucci M., Queiroz-Telles F., Alvarado-Matute T., Tiraboschi I.N., Cortes J., Zurita J., Guzman-Blanco M., Santolaya M.E., Thompson L., Sifuentes-Osornio J. (2013). Epidemiology of candidemia in Latin America: A laboratory-based survey. PLoS ONE.

[6] Cornely O.A., Bassetti M., Calandra T., Garbino J., Kullberg B.J., Lortholary O., Meersseman W., Akova M., Arendrup M.C., Arikan-Akdagli S. (2012). ESCMID* guideline for the diagnosis and management of Candida diseases 2012: Non-neutropenic adult patients. Clin. Microbiol. Infect..

[7] Colombo A.L., Guimarães T., Camargo L.F., Richtmann R., Queiroz-Telles Fd Salles M.J., Cunha C.A., Yasuda M.A., Moretti M.L., Nucci M. (2013). Brazilian guidelines for the management of candidiasis-a joint meeting report of three medical societies: Sociedade Brasileira de Infectologia, Sociedade Paulista de Infectologia and Sociedade Brasileira de Medicina Tropical. Braz. J. Infect. Dis..

[8] Pappas P.G., Kauffman C.A., Andes D.R., Clancy C.J., Marr K.A., Ostrosky-Zeichner L., Reboli A.C., Schuster M.G., Vazquez J.A., Walsh T.J. (2016). Clinical Practice Guideline for the Management of Candidiasis: 2016 Update by the Infectious Diseases Society of America. Clin. Infect. Dis..

[9] Vena A., Bouza E., Valerio M., Padilla B., Paño-Pardo J.R., Fernández-Ruiz M., Díaz Martín A., Salavert M., Mularoni A., Puig-Asensio M. (2017). Candidemia in non-ICU surgical wards: Comparison with medical wards. PLoS ONE.

[10] Ohki S., Shime N., Kosaka T., Fujita N. (2020). Impact of host- and early treatment-related factors on mortality in ICU patients with candidemia: A bicentric retrospective observational study. J. Intensive Care.

[11] Colombo A.L., Guimarães T., Sukienik T., Pasqualotto A.C., Andreotti R., Queiroz-Telles F., Nouér S.A., Nucci M. (2014). Prognostic factors and historical trends in the epidemiology of candidemia in critically ill patients: An analysis of five multicenter studies sequentially conducted over a 9-year period. Intensive Care Med..

[12] Agnelli C., Guimarães T., Sukiennik T., Lima P.R.P., Salles M.J., Breda G.L., Queiroz-Telles F., Chaves Magri M.M., Mendes A.V., Camargo L.F.A. (2023). Prognostic Trends and Current Challenges in Candidemia: A Comparative Analysis of Two Multicenter Cohorts within the Past Decade. J. Fungi.

[13] Calà C., Fontana I., Di Carlo P., Mascarella C., Fasciana T., Reale S., Sergi C., Giammanco A. (2020). Candida parapsilosis Infection: A Multilocus Microsatellite Genotyping-Based Survey Demonstrating an Outbreak in Hospitalized Patients. Ann. Clin. Lab. Sci..

[14] Hamburger F.G., Gales A.C., Colombo A.L. (2024). Systematic Review of Candidemia in Brazil: Unlocking Historical Trends and Challenges in Conducting Surveys in Middle-Income Countries. Mycopathologia.

[15] Colombo A.L., Nucci M., Park B.J., Nouér S.A., Arthington-Skaggs B., da Matta D.A., Warnock D., Morgan J., Brazilian Network Candidemia Study (2006). Epidemiology of Candidemia in Brazil: A Nationwide Sentinel Surveillance of Candidemia in Eleven Medical Centers. J. Clin. Microbiol..

[16] Guimarães T., Nucci M., Mendonça J.S., Martinez R., Brito L.R., Silva N., Moretti M.L., Salomão R., Colombo A.L. (2012). Epidemiology and predictors of a poor outcome in elderly patients with candidemia. Int. J. Infect. Dis..

[17] Doi A.M., Pignatari A.C.C., Edmond M., Marra A.R., Camargo L.F.A., Siqueira R.A., Da Mota V.P., Colombo A.L. (2016). Epidemiology and Microbiologic Characterization of Nosocomial Candidemia from a Brazilian National Surveillance Program. PLoS ONE.

[18] Bassetti M., Giacobbe D.R., Vena A., Trucchi C., Ansaldi F., Antonelli M., Adamkova V., Alicino C., Almyroudi M.P., Atchade E. (2019). Incidence and outcome of invasive candidiasis in intensive care units (ICUs) in Europe: Results of the EUCANDICU project. Crit. Care.

[19] Vaquero-Herrero M.P., Ragozzino S., Castaño-Romero F., Siller-Ruiz M., Sánchez González R., García-Sánchez J.E., García-García I., Marcos M., Ternavasio-de la Vega H.G. (2017). The Pitt Bacteremia Score, Charlson Comorbidity Index and Chronic Disease Score are useful tools for the prediction of mortality in patients with Candida bloodstream infection. Mycoses.

[20] Bassetti M., Giacobbe D.R., Agvald-Ohman C., Akova M., Alastruey-Izquierdo A., Arikan-Akdagli S., Azoulay E., Blot S., Cornely O.A., Cuenca-Estrella M. (2024). Invasive Fungal Diseases in Adult Patients in Intensive Care Unit (FUNDICU): 2024 consensus definitions from ESGCIP, EFISG, ESICM, ECMM, MSGERC, ISAC, and ISHAM. Intensive Care Med..

[21] Puig-Asensio M., Pemán J., Zaragoza R., Garnacho-Montero J., Martín-Mazuelos E., Cuenca-Estrella M., Almirante B., Prospective Population Study on Candidemia in Spain (CANDIPOP) Project, Hospital Infection Study Group (GEIH), Medical Mycology Study Group (GEMICOMED) of the Spanish Society of Infectious Diseases and Clinical Microbiology (SEIMC) (2014). Impact of therapeutic strategies on the prognosis of candidemia in the ICU. Crit. Care Med..

[22] Agnelli C., Valerio M., Bouza E., Guinea J., Sukiennik T., Guimarães T., Queiroz-Telles F., Muñoz P., Colombo A.L. (2021). Prognostic factors of Candida spp. bloodstream infection in adults: A nine-year retrospective cohort study across tertiary hospitals in Brazil and Spain. Lancet Reg. Health Am..

[23] Martin-Loeches I., Antonelli M., Cuenca-Estrella M., Dimopoulos G., Einav S., De Waele J.J., Garnacho-Montero J., Kanj S.S., Machado F.R., Montravers P. (2019). ESICM/ESCMID task force on practical management of invasive candidiasis in critically ill patients. Intensive Care Med..

[24] Peçanha-Pietrobom P.M., Colombo A.L. (2020). Mind the gaps: Challenges in the clinical management of invasive candidiasis in critically ill patients. Curr. Opin. Infect. Dis..

[25] Araujo J.M., de Almeida Junior J.N., Magri M.M.C., Costa S.F., Guimarães T. (2024). Guideline Adherence and Outcomes of Patients with Candidemia in Brazil. J. Fungi.

[26] Andes D. (2019). Has the Optimal Therapy for Invasive Candidiasis Now Been Defined?. Clin. Infect. Dis..

[27] Andes D.R., Safdar N., Baddley J.W., Playford G., Reboli A.C., Rex J.H., Sobel J.D., Pappas P.G., Kullberg B.J., Mycoses Study Group (2012). Impact of treatment strategy on outcomes in patients with candidemia and other forms of invasive candidiasis: A patient-level quantitative review of randomized trials. Clin. Infect. Dis..

[28] Reboli A.C., Rotstein C., Pappas P.G., Chapman S.W., Kett D.H., Kumar D., Betts R., Wible M., Goldstein B.P., Schranz J. (2007). Anidulafungin versus fluconazole for invasive candidiasis. N. Engl. J Med..

[29] Reboli A.C., Shorr A.F., Rotstein C., Pappas P.G., Kett D.H., Schlamm H.T., Reisman A.L., Biswas P., Walsh T.J. (2011). Anidulafungin compared with fluconazole for treatment of candidemia and other forms of invasive candidiasis caused by Candida albicans: A multivariate analysis of factors associated with improved outcome. BMC Infect. Dis..

[30] Demir K.K., Butler-Laporte G., Del Corpo O., Ekmekjian T., Sheppard D.C., Lee T.C., Cheng M.P. (2021). Comparative effectiveness of amphotericin B, azoles and echinocandins in the treatment of candidemia and invasive candidiasis: A systematic review and network meta-analysis. Mycoses.

[31] Braga P.R., Cruz I.L., Ortiz I., Barreiros G., Nouér S.A., Nucci M. (2018). Secular trends of candidemia at a Brazilian tertiary care teaching hospital. Braz. J. Infect. Dis..

[32] Bassetti M., Giacobbe D.R., Vena A., Wolff M. (2019). Diagnosis and Treatment of Candidemia in the Intensive Care Unit. Semin. Respir. Crit. Care Med..

[33] Yan T., Li S.L., Ou H.L., Zhu S.N., Huang L., Wang D.X. (2020). Appropriate Source Control and Antifungal Therapy are Associated with Improved Survival in Critically Ill Surgical Patients with Intra-abdominal Candidiasis. World J. Surg..

